# Regioselective Transfer Hydrogenation of Substituted Oxiranes with Alcohols Using MgO as the Catalyst

**DOI:** 10.3390/molecules30214212

**Published:** 2025-10-28

**Authors:** Marek Gliński, Patrycja Waniek

**Affiliations:** Faculty of Chemistry, Warsaw University of Technology, Noakowskiego 3, 00664 Warsaw, Poland

**Keywords:** catalytic transfer hydrogenation, regioselectivity, oxiranes, alcohols, magnesium oxide catalyst

## Abstract

An excellent regioselectivity of vapor-phase catalytic transfer hydrogenation (CTH) of substituted oxiranes (methyl-, *n*-butyl-, and phenyloxirane) with alkanols (EtOH, 2-PrOH or 2-PeOH) as hydrogen donors in the presence of magnesium oxide as a catalyst was attained. In the vapor phase, all of these oxiranes as well as 1,2-epoxycyclohexane were hydrogenated. Moreover, it was found that primary alcohols were always the main products of CTH of methyl-, *n*-butyl-, and phenyloxirane with very good regioselectivity towards the alcohols: 93, 73, and 100%, respectively. It was shown that vapor-phase CTH of methyloxirane with 2-pentanol led to three products, two regioisomeric propanols, (1-PrOH and 2-PrOH), and also 1-(2-pentyloxy)-2-propanol. Their yields were 48%, 4%, and 35%, respectively. Two regioisomeric hexanols (1-HeOH, 54% yield and 2-HeOH, 20% yield) and 2-hexanone (14% yield) were found as products of the CTH of *n*-butyloxirane with 2-propanol as the hydrogen donor. For vapor-phase CTH of phenyloxirane, only 2-phenylethanol (95% yield) was observed together with minor amounts of phenylacetaldehyde. In vapor-phase CTH of 1,2-epoxycyclohexane, the presence of the transfer hydrogenation products (2-cyclohexenone, cyclohexanol, and cyclopentylmethanol) as well as isomerization products (cyclohexanone, 2-cyclohexenol, and 3-cyclohexenol) were found. It was noted that at 623 K, the yields of the former products were 18, 22, and 14%, respectively. Liquid-phase CTH of *n*-butyl- or phenyloxirane with 2-pentanol (b.p. 392 K) was unsuccessful.

## 1. Introduction

Catalytic transfer hydrogenation (CTH) is an alternative method of hydrogenation of organic compounds. In this method, instead of using pressurized gaseous dihydrogen as the reducing agent, organic compounds that are capable of donating hydrogen are applied. Hence, in this method, hydrogenation occurs under normal pressure and at moderate temperatures. In recent years, CTH has been a frequently explored research topic [1,2,3,4]. According to the data contained in a 2023 review article, over 286,000 articles on this topic have been published over the last two decades [1]. It should be noted that the vast majority of these works concern the use of homogeneous catalysts in the form of transition metal complexes (Co, Rh, Ru, Os, Ir, Rh, Fe, Pd, and Ni). Numerous comprehensive reviews have examined the activities of these homogeneous catalysts involved in transfer hydrogenation [5,6,7,8]. Various heterogeneous catalysts such as transition metals deposited on inorganic supports, magnesium oxide, hydrotalcites, hydrous zirconia, supported ZrO_2_, zeolites, and grafted lanthanide alkoxides have been tested in the CTH of various organic compounds [8,9,10,11,12]. The transfer hydrogenation of carbonyl compounds was of particular interest due to its high chemoselectivity toward carbonyl group reduction and its mild reaction conditions. The catalytic hydrogenation of epoxides was not limited to the use of pressurized dihydrogen. In the last few years, different sources other than dihydrogen were also explored. Yu et al. described Pd nanoparticles microencapsulated in urea as an efficient and recyclable catalyst for the hydrogenolysis of epoxides using HCOOH/Et_3_N as the hydrogen donor [13]. Werner et al. disclosed cobalt pincer catalyst for the ring opening of epoxides via transfer hydrogenation using NH_3_BH_3_ (ammonia borane) as the hydrogen source. Notably, a catalytic amount of Er(OTf)_3_ was used as a highly efficient isomerization catalyst which converted epoxides into carbonyl intermediates, and further transfer hydrogenation led to the formation of alcohols [14]. To date, we have investigated hydrogen transfer to carbonyl compounds, both ketones [15,16,17] and aldehydes [18,19,20], in a number of studies. The results obtained confirmed the usefulness of catalytic transfer hydrogenation in the synthesis of a range of alcohols over several oxides [21]. Among its many advantages was its chemoselective or regioselective reduction. The exceptionally high activity of the MgO as the catalyst has prompted us to perform additional studies with this oxide. In the present work, we have undertaken a novel topic of hydrogen transfer from alcohols to monosubstituted oxiranes, mainly to determine the regioselectivity of the hydrogenolysis reaction of oxiranes rings and to investigate their reactivity in CTH in the presence of magnesium oxide as the catalyst. To the authors’ knowledge, such studies have not yet been performed with these types of compounds.

Oxiranes and alcohols are fundamental compounds used as an industrial feedstock for the production of fine and bulk chemicals [22]. Alcohols are widely used as industrial solvents, fuels, and pharmaceuticals. Many different methods of obtaining alcohols are known, the main ones are hydrogenation of carbonyl compounds [23,24,25,26] and hydrogenation of oxiranes [27,28,29,30]. Traditional reductions in oxiranes were achieved using strong reducing reagents such as LiAlH_4_ and NaBH_4_ [31,32,33]. Unfortunately, these stoichiometric metal hydride reactions suffer from selectivity issues and generate copious metal sludge as waste. In the case of hydrogenation of carbonyl compounds, the position of the carbonyl group in ketones or the formyl group in aldehydes determines the position and order of the alcohol obtained [9,15,16,34]. Hydrogenation, or rather hydrogenolysis, of the substituted oxiranes results in the formation of two regioisomeric alcohols, depending on which carbon–oxygen bond is broken. The problem of regioselectivity in the hydrogenation of the substituted oxiranes has been the subject of several studies in the past [29,30,35,36,37,38]. The focus has been on collecting data on the influence of the structure of oxirane, type of its substituents, type of catalyst, and reaction conditions on the composition of the post-reaction mixture. The heterogeneous catalysts used were mainly those containing palladium supported on activated carbon, complexed with ethylenediamine [39], on Fe_2_O_3_ nanoparticles [40], as well as palladium nanoparticles in the form of a suspension in water as the dispersing phase [41,42]. The iridium pincer catalyst in the presence of triflic acid is also a hydrogenation catalyst with moderate activity [36]. In the presence of these catalysts, the hydrogenation of epoxides such as phenyloxirane resulted mainly in the formation of primary alcohol, i.e., 2-phenylethanol. Depending on the catalyst type and reaction conditions, yields of this alcohol of up to 97% have been reported [41]. The use of homogeneous catalysts in the hydrogenation of epoxides allowed for a much easier control of the reaction regioselectivity. For example, Ikarya et al., in the presence of a complex catalyst, namely Cp*RuCl(cod), 2-(diphenylphosphino) ethylamine, and KOH, obtained a mixture containing predominantly secondary alcohol (1-phenylethanol) in the hydrogenation of phenyloxirane. With an overall conversion of 79%, the ratio of the two alcohols as products was 89% (1-PhEtOH) to 11% (2-PhEtOH) [43]. Similarly, in the case of using a Ru-MACHO pincer catalyst and *t*-BuOK as a strong base, the hydrogenation of phenyloxirane led to the predominance of 1-PhEtOH in the reaction products [30]. High regioselectivity to 2-PhEtOH (99%) was observed in the hydrogenation of phenyloxirane in the presence of an iron carbonyl complex catalyst [38] and a Co(BF_4_)·6H_2_O/triphos catalyst with the addition of TFA (94%) [44].

The selective synthesis of one of the two possible regioisomers of alcohols is a difficult task to achieve. A key challenge in the selective ring opening of oxiranes is the control of regioselectivity. In the present work, a thorough investigation of the reactivity and regioselectivity of selected oxiranes in hydrogen transfer reduction by alcohols using magnesium oxide as the catalyst either in liquid or in vapor phase has been performed. Through the conducted studies, we obtained answers to questions related to the reactivity of oxiranes and the possibility of effective hydrogen transfer to their molecules in the liquid phase at temperatures close to 393 K, using 2-pentanol as a hydrogen donor. Another key topic was the explanation of the regioselectivity of the ring opening of monosubstituted oxiranes in which the substituents were methyl, *n*-butyl, and phenyl groups. For 1,2-epoxycyclohexane, the fourth oxirane selected for this study, we expected an answer to the question of whether the system of two fused rings and, additionally, the specific structure of the three-membered oxirane ring would allow for its highly selective reduction only to cyclohexanol.

## 2. Results

### 2.1. Characterization of a Catalyst

Since the same batch of magnesium oxide was the subject of research presented in our recent publications and the present work, for an extensive characterization the reader is referred to [20,21]. In short, the catalyst used in this study can be described as follows:

- The specific surface area of MgO calcined at 873 K was 100 m^2^ g^−1^.
- The pore volume of the oxide was 0.529 cm^3^ g^−1^.
- The X-ray diffraction studies revealed the presence of a single, periclase phase.
- The crystallite size of 12 nm was determined based on the diffraction pattern analysis, 2θ = 42.9° (200).
- The range of basic site strengths as indicated by the Hammett indicator tests was 7.2 ≤ H_−_ < 33.0, whereas none of the basic Hammett indicators changed their color in its presence (none were protonated). Therefore, the tested MgO had a wide range of strengths of basic sites on its surface, up to superbasic ones with strength H_−_ ≤ 26.5 (4-chloroaniline as the indicator), and was devoid of acidic sites in the studied range.

### 2.2. Activity Measurement Results

In the current study on catalytic transfer hydrogenation, four substituted oxiranes were used as hydrogen acceptors, including methyl-, *n*-butyloxirane, phenyloxirane, and one oxirane with a more complex structure, namely 1,2-epoxycyclohexane, whose systematic name is 7-oxabicyclo[4.1.0]heptane, containing two fused rings, one of which is six-membered and the other is a three-membered ring. Three aliphatic alcohols, namely ethanol, 2-propanol, and 2-pentanol were used as hydrogen donors, and magnesium oxide was applied as the catalyst in both liquid- and vapor-phase modes of reaction.

#### 2.2.1. Vapor-Phase Transfer Hydrogenation of Methyloxirane

**Scheme 1:**
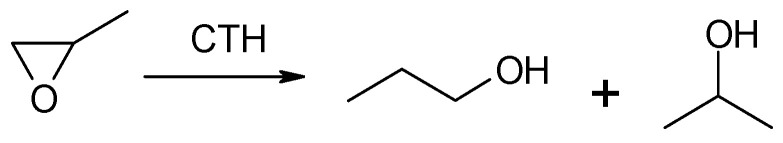
Methyloxirane and two regioisomeric alcohols (1-propanol and 2-propanol) formed during its reduction using MgO as the catalyst.

**Figure 1:**
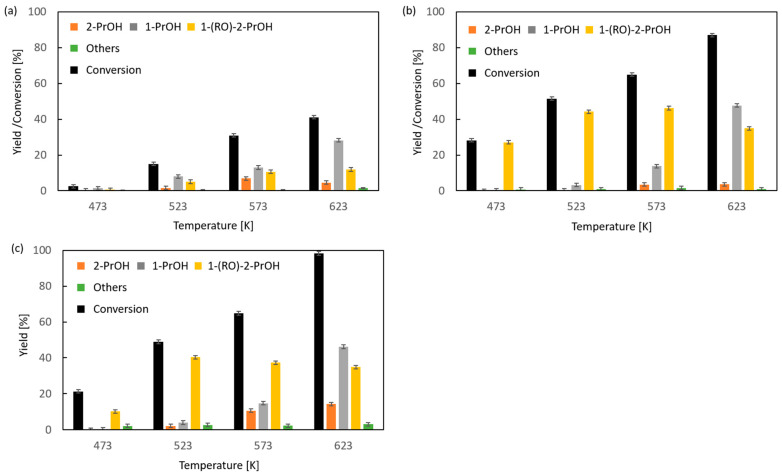
Influence of donor–acceptor molar ratio on the composition of the post-reaction mixture of vapor-phase transfer hydrogenation of methyloxirane with 2-pentanol in the presence of magnesium oxide as the catalyst: (**a**) D/A = 3, (**b**) D/A = 6, and (**c**) D/A = 9.

Due to the very low boiling point of methyloxirane (b.p. 308 K) and because 2-propanol is the expected reduction product of this oxirane, no studies on the transfer hydrogenation in the liquid or vapor phase with the participation of 2-propanol as the hydrogen donor were made. Studies were carried out on CTH in vapor phase using 2-pentanol as a donor at a variable donor–acceptor molar ratio of 3, 6, and 9 (Figure 1). The transfer hydrogenation from 2-pentanol to methyloxirane is shown in Scheme 1.

**Scheme 2:**
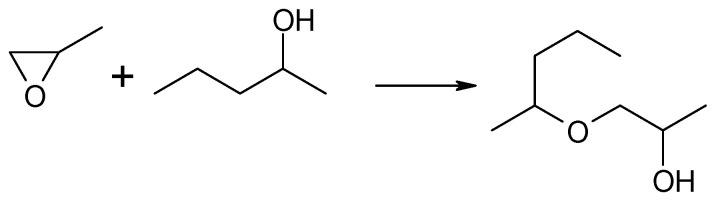
Reaction of methyloxirane with 2-pentanol in the presence of MgO as the catalyst.

In this reaction, two regioisomeric propanols were formed. Acetone, a product of methyloxirane isomerization, was not observed in the post-reaction mixture. Instead, a significant contribution to the reaction products was noted: the addition product of 2-pentanol to methyloxirane, the formation of which resulted in the opening of the oxirane ring and the incorporation of the 2-pentanol molecule into the new structure of the formed alkoxy alcohol, i.e., 1-(2-pentyloxy)propan-2-ol, as illustrated in Scheme 2.

It was found that for the 2-pentanol–methyloxirane mixture with a molar ratio of 3 (Figure 1a), the conversion increased with temperature reaching the highest value of 41%. In the entire studied reaction temperature range (473–623 K), oxirane reduction took place, and the primary alcohol, i.e., 1-propanol, was the main product. The highest yield of this alcohol (28%) was recorded at a temperature of 623 K. The highest yield of 2-propanol (7%) was achieved at a temperature of 573 K. The molar ratio of regioisomeric alcohols (1-PrOH/2-PrOH) was in the range of (2–6):1. The fraction of 1-(2-pentyloxy)-2-propanol increased with the reaction temperature, reaching a maximum yield of 12%. An increase in the molar ratio to 6 resulted, firstly, in a significant increase in conversion of up to 87% at 623 K (Figure 1b). Secondly, a large increase in the yield of 1-PrOH, up to 48%, was noted. Thirdly, a nearly four-fold increase in the yield of 1-(2-pentyloxy)-2-propanol was observed. In this case the maximum (46%) already occurred at 573 K. A further increase in the donor–acceptor ratio (D/A = 9) resulted in an increase in the conversion value to 98% at 623 K and an increase in the yield of 2-PrOH to 14% (Figure 1c). At 623 K, the highest yield of 1-PrOH was recorded (46%), which gives a 1-PrOH/2-PrOH ratio of 3.25. The maximum yield of 1-(2-pentyloxy)-2-propanol was 40%, which was already noted at 573 K, but it was lower than the corresponding yield of this compound obtained at a ratio of D/A = 6.

#### 2.2.2. Vapor-Phase Transfer Hydrogenation of n-Butyloxirane

**Scheme 3:**
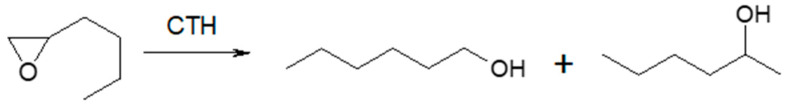
*n*-Butyloxirane and two regioisomeric alcohols (1-hexanol and 2-hexanol) formed during its reduction in the presence of MgO as the catalyst.

In vapor-phase transfer hydrogenation of *n*-butyloxirane with 2-propanol or ethanol (Table 1) in the entire reaction temperature range (523–673 K), the formation of *n*-butyloxirane reduction products in the form of regioisomeric alcohols, 1-hexanol, and 2-hexanol was observed (Scheme 3).

Their yields increased with temperature, with the primary alcohol always being the dominant product. The regioselectivity of the reaction towards 1-hexanol was in the range of 63–73%. The presence of 2-hexanone, a product of oxirane isomerization, was observed in the post-reaction mixtures starting from a temperature of 623 K, regardless of the hydrogen donor (2-propanol, ethanol) or the donor–acceptor molar ratio (3–9:1). The ketone yield increased with the reaction temperature, reaching the highest value (15%) at 673 K. Ethanol was a significantly less reactive hydrogen donor than 2-propanol. In its presence, a conversion of only 44% was observed at 673 K, with reduced yields of both alcohols. However, the yield of 2-hexanone did not change. Unlike for methyloxirane, the presence of the corresponding alkoxy alcohol was not observed in the post-reaction mixture with *n*-butyloxirane and both hydrogen donors.

In order to determine potential changes in the activity of the MgO catalyst over time, a time-on-stream test was performed for the 2-propanol–*n*-butyloxirane system at 673 K (Table 2). It was found that in the time interval of 0.5–6 h, the conversion decreased from the initial value of 87 to 68%, whereas the relationships between the yields of individual reaction products remained unchanged. The test was repeated with the spent catalyst, where a further reduction in conversion to 58% was observed after another 6 h of reaction, presumably caused by the deposition of heavy side products on the surface of MgO.

Attempts to reduce *n*-butyloxirane with 2-pentanol in the liquid phase (D/A = 6, T_R_ = 392 K) within 6 h in the presence of MgO were unsuccessful due to the lack of reactivity of oxirane under these reaction conditions.

#### 2.2.3. Vapor-Phase Transfer Hydrogenation of Phenyloxirane

**Scheme 4:**
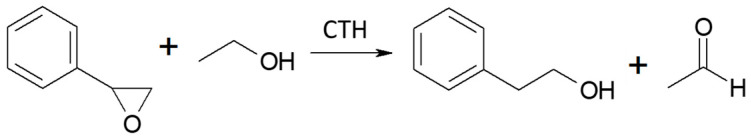
CTH of styrene oxide with ethanol in the presence of MgO as the catalyst.

**Figure 2:**
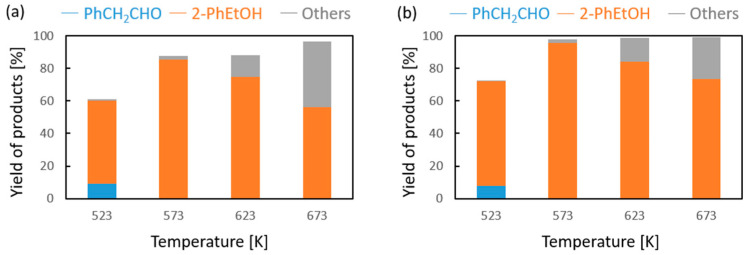
Influence of the donor–acceptor molar ratio on the composition of the post-reaction mixture of ethanol and phenyloxirane in the presence of magnesium oxide as the catalyst: (**a**) D/A = 3 and (**b**) D/A = 6.

**Figure 3:**
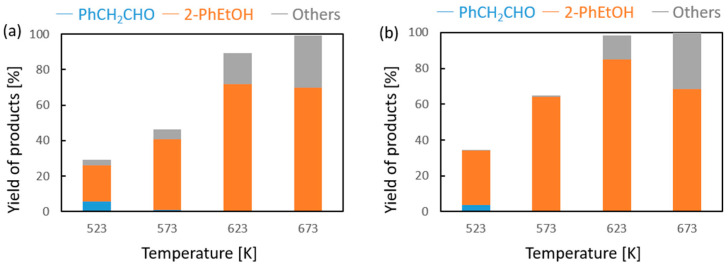
Influence of the donor–acceptor molar ratio on the composition of the post-reaction mixture of 2-propanol and phenyloxirane in the presence of magnesium oxide as the catalyst: (**a**) D/A = 3 and (**b**) D/A = 6.

Vapor-phase catalytic tests were performed using styrene oxide as the hydrogen acceptor and two donors, ethanol and 2-propanol, in the presence of MgO as the catalyst. The results are summarized in Figure 2 and Figure 3, respectively. Two of the reaction products were phenylacetaldehyde and 2-phenylethanol. Moreover, heavy condensation products were also found in the post-reaction mixture, especially at higher temperatures. The former was an isomerization product of styrene oxide and was present in the reaction products only at the two lowest reaction temperatures (523 and 573 K) regardless of the type of hydrogen donor used (ethanol, 2-propanol) and the donor–acceptor molar ratio. Its yield did not exceed 10% and decreased rapidly with reaction temperature. 2-Phenylethanol was the only product of styrene oxide reduction; the presence of the second regioisomer, i.e., 1-phenylethanol, was not observed in any of the post-reaction mixtures. The highest yield of 2-phenylethanol of 95% was already obtained at 573 K in the presence of ethanol at a D/A molar ratio of 6. There was also no formation of 2-phenyl-2-(2-propyloxy) which is the product of addition of the hydrogen donor molecule to styrene oxide. The transfer hydrogenation from ethanol to styrene oxide is shown in Scheme 4.

**Scheme 5:**
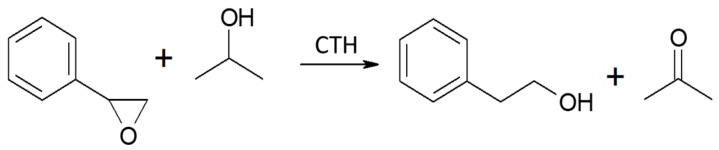
CTH of styrene oxide with 2-propanol in the presence of MgO as the catalyst.

The transfer hydrogenation from 2-propanol to styrene oxide is shown in Scheme 5.

It was found that in the case of hydrogen transfer to styrene oxide in the presence of MgO as the catalyst, 2-propanol is a significantly less reactive hydrogen donor than ethanol (Figure 3). Both the conversion and yield of 2-phenylethanol are significantly lower than those achieved with ethanol. This is in clear contrast to the reactivity of 2-propanol observed by us previously in the case of transfer hydrogenation of many types of carbonyl compounds with alcohols in the presence of MgO as a catalyst [16,17]. A similar decrease in the reactivity of 2-propanol as the hydrogen donor compared to the reactivity of ethanol was observed only in the transfer hydrogenation of acrolein and some of its derivatives [17]. In our previous work, it was shown that the reactivity of alcohols in reaction with acrolein is determined by the order of the alcohol [18]. Primary alcohols (e.g., ethanol, 1-butanol) show much higher reactivity than secondary ones (2-propanol, 2-octanol). The reason for this behavior of secondary alcohols is not yet explained, although a thermodynamic analysis of the hydrogen transfer reaction from ethanol and 2-propanol as hydrogen donors to carbonyl compounds clearly showed that the dehydrogenation of the secondary alcohol is thermodynamically favored over that of the primary alcohol. At a temperature of 623 K, the difference in the free Gibbs energy value (ΔG) is 12.06 kJ/mol in favor of the secondary alcohol (2-propanol) [16]. Moreover, the influence of a steric hindrance present during hydrogen transfer from the adsorbed donor molecule to the neighboring acceptor molecule can be excluded, because the strongest decrease in the reactivity of 2-propanol was noted for acrolein, which has no steric hindrances in the proximity of the formyl group.

**Scheme 6:**
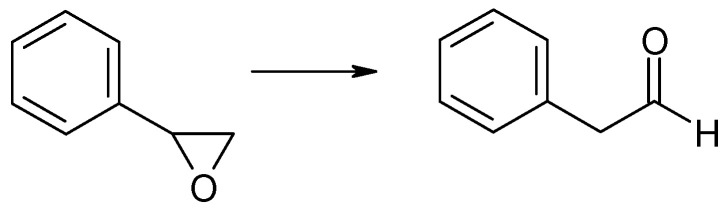
The isomerization reaction of styrene oxide in the presence of MgO as the catalyst.

**Scheme 7:**
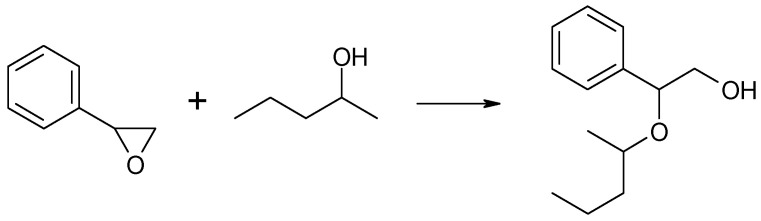
The formation of 2-phenyl-2-(2-pentyloxy) ethanol from styrene oxide and 2-pentanol in the presence of MgO as the catalyst.

Similarly to *n*-butyloxirane, phenyloxirane (styrene oxide) did not undergo CTH in the liquid phase with 2-pentanol as the hydrogen donor in the presence of MgO as the catalyst despite the reaction mixture being heated to boiling (392 K) for 6 h. However, two other transformations took place in the reaction mixture, i.e., the isomerization of styrene oxide combined with the opening of the oxirane ring to form phenylacetaldehyde, and the addition of 2-pentanol to the oxirane molecule combined with the formation of an alkoxy alcohol-2-phenyl-2-(2-pentyloxy) ethanol. Both transformations are illustrated in the schemes below (Scheme 6 and Scheme 7).

A 35% conversion of oxirane was observed after 6 h of reaction, with the fraction of phenylacetaldehyde being practically constant over time (30–360 min) and amounting to 10–12% (Table 3). After 60 min, the main reaction product was 2-phenyl-2-(2-pentyloxy) ethanol, i.e., the product of the addition of 2-pentanol to styrene oxide. Its yield increased with reaction time. After 30 and 360 min, the yields were 5% and 23%, respectively. No formation of 2-phenylethanol, i.e., the product of hydrogen transfer from 2-pentanol to styrene oxide, was observed during the 360 min-long reaction.

#### 2.2.4. Vapor-Phase Transfer Hydrogenation of 1,2-Epoxycyclohexane

**Scheme 8:**
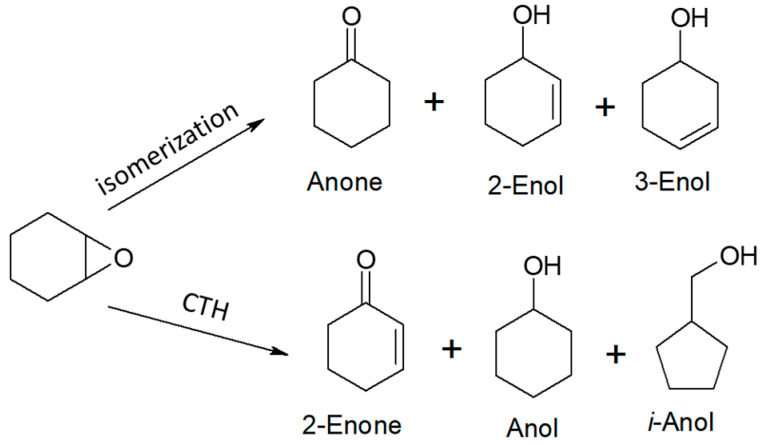
Products formed during transformation of 1,2-epoxycyclohexane in the presence of ethanol or 2-propanol and magnesium oxide as the catalyst.

1,2-Epoxycyclohexane was the fourth and last acceptor whose reactivity and transformations were studied in the vapor-phase mode in the hydrogen transfer reaction from ethanol and 2-propanol as hydrogen donors. A number of products were observed to be formed from both the hydrogen transfer and isomerization reactions (Scheme 8). The top row of compounds in Scheme 8 contains the isomerization products of 1,2-epoxycyclohexane and the bottom one contains the products of its hydrogenation/dehydrogenation. In addition to the above-mentioned products, identified as components of post-reaction mixtures, the presence of ethyl- or isopropylphenol derivatives (with yield up to 0.3%) was also found in some mixtures. The results indicate that among the potential substrates in the transformation leading to Anol, it is the Anone and not 2-Enol which is the key compound in its synthesis. This is confirmed by the results of previous studies of CTH taken from the literature in which Anone (cyclohexanone) is a very reactive hydrogen acceptor, unlike 2-Enol.

**Figure 4:**
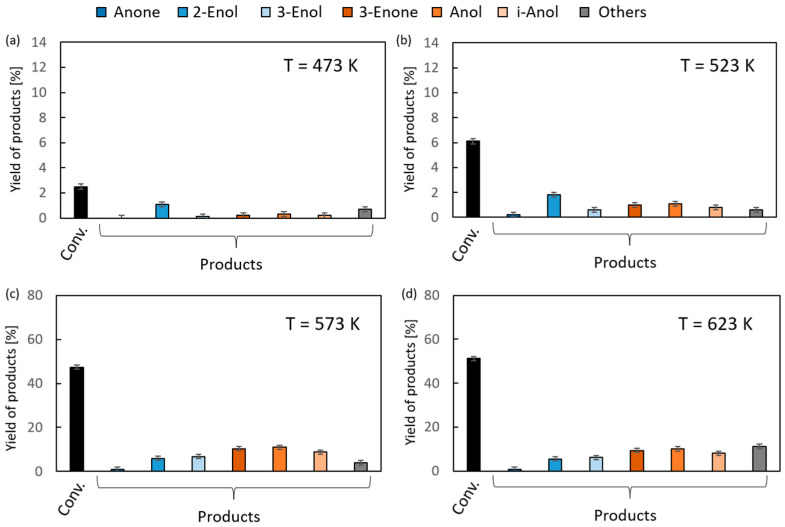
The results of vapor-phase transfer hydrogenation of 1,2-epoxycyclohexane with ethanol in the presence of MgO as a catalyst (D/A = 6) at different reaction temperatures: (**a**) 473 K, (**b**) 523 K, (**c**) 573 K and (**d**) 623 K.

The results of the vapor-phase transfer hydrogenation from ethanol to 1,2-epoxycyclohexane in the presence of magnesium oxide as the catalyst are given in Figure 4. For clarity of the figure, the first column (black) in each chart corresponds to the overall conversion of 1,2-epoxycyclohexane, the first three products (blue) are the isomerization reaction products (Anone, 2-Enol, and 3-Enol, respectively), and the next three (orange) are transfer hydrogenation products (2-Enone, Anol, and *i*-Anol, respectively) of 1,2-epoxycyclohexane. In the reaction temperature range of 523–573 K, very low conversions (up to 6%) were observed. An increase of up to 51% was noted at the highest temperature (673 K). Above 573 K, the reaction products were dominated by hydrogen transfer products. Their yields were similar, ranging from 8% to 11%. The relatively high yield of *i*-Anol (cyclopentylmethanol) (8–9%) is noteworthy, the formation of which is associated with the contraction of the six-membered ring of the substrate. The formation of this compound with a yield of 39% as the main product of the hydrogenation reaction of 1,2-epoxycyclohexane in the presence of a homogeneous iridium catalyst was reported in the literature [35]. Among the isomerization products, the main products were 2- and 3-cyclohexenols, and their maximum yield was 5–7%, whereas the yield of the third one, cyclohexanone, did not exceed 1%.

**Figure 5:**
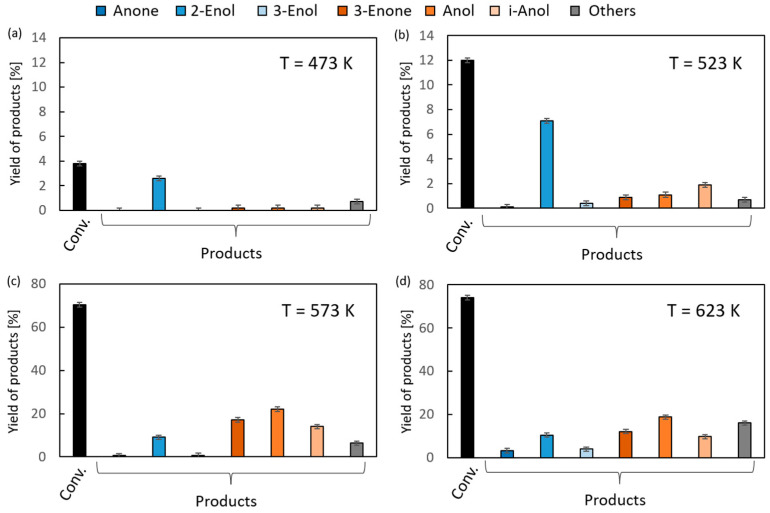
The results of vapor-phase transfer hydrogenation of 1,2-epoxycyclohexane with 2-propanol in the presence of MgO as a catalyst (D/A = 6) at different reaction temperatures: (**a**) 473 K, (**b**) 523 K, (**c**) 573 K and (**d**) 623 K.

A significantly higher reactivity in the reaction with 1,2-epoxycyclohexane was found in the case of 2-propanol as the hydrogen donor (Figure 5). Conversions of 70–74% were recorded in the temperature range of 623–673 K. The total yield of hydrogen transfer reaction products was much higher than that of isomerization reaction products, similar to the reaction using ethanol as the donor (Figure 4). It was shown that 2-Enol was the main product of the substrate isomerization. At a temperature of 673 K, its yield was 10%. It is noteworthy that the yield of 3-Enol is lower than in the reaction with ethanol, which is 4% (673 K). The highest yields of hydrogen transfer reaction products were recorded at a temperature of 623 K. The yield of Anol, the main reaction product, reached 22%.

It was found that in the catalytic transfer hydrogenation of oxiranes by alcohols carried out in the presence of MgO as the catalyst, the reducing agent (H^−^) preferentially attacks the more substituted carbon atom of the oxirane. As a result, the bond between the carbon atom and the oxygen atom is broken and the primary alcohol is formed. The preference of this attack depends on the reaction conditions and, above all, on the structure of the oxirane. The presence of an electron-withdrawing substituent in the form of a phenyl group, as in phenyloxirane, results in the attack of the reducing agent only on this carbon atom, resulting in the formation of 2-phenylethanol with 100% regioselectivity. The presence of an electron-donating group (e.g., methyl or *n*-butyl) enables the attack of the reducing agent on both carbon atoms of the oxirane and the formation of primary and secondary alcohols, with a clear preference for the attack on the higher substituted carbon atom, and thus, the regioselectivity to the primary alcohol is above 70% for both methyloxirane and *n*-butyloxirane.

## 3. Materials and Methods

### 3.1. Catalyst and Its Characterization

Pure magnesium oxide (MgO) was synthesized from commercial magnesium oxide (purum p.a., Reachim, Chişinåu, Moldova), as described in our previous work [45]. A fraction of 0.16–0.40 mm grains of the resulting Mg(OH)_2_ was calcined at 873 K in a stream of air for 1 h and for 5 h in a stream of dry nitrogen. After cooling, the oxide was stored under nitrogen in a Schlenk-type container.

The following measurements and methods have been used to characterize the catalyst: the specific surface area measurement, XRD analysis, Scanning Electron Microscopy Coupled with Energy-Dispersive X-ray Spectroscopy (SEM-EDX), and TGA and DTA measurements. Detailed methods of their use, along with a description of the measurement devices, have been described in our previous publications [20,21]. The strength of the surface acid–base sites of MgO was determined by the Hammett method using a sequence of solutions (0.1%) of the following indicators (in parentheses, values of pK_a_ or pK_BH_+ of indicators are given): crystal violet (0.8), methyl red (4.8), bromothymol blue (7.2), phenolphthalein (9.3), 2,4-dinitroaniline (15.0), 4-nitroaniline (18.4), diphenylamine (22.3), 4-chloroaniline (26.5), and triphenylmethane (33.0) in anhydrous toluene [19]. The probed scale of acidity and basicity were, therefore, 0.8 ≤ H_0_ ≤ 4.8 and 7.2 ≤ H_−_ ≤ 33, respectively.

### 3.2. Reagents

#### 3.2.1. Hydrogen Donors

Three aliphatic alcohols: ethanol (anhydrous 99.8%, POCh, Gliwice, Poland), 2-propanol (puriss., POCh, Gliwice, Poland), and 2-pentanol (98%, Aldrich (Poznań, Poland) were purchased. Ethanol was dried according to the Lund-Bjerrum method with magnesium and iodine and distilled under normal pressure in dry nitrogen atmosphere. The purification of the other two alcohols was described in our previous work [19]. The final purities (GC) of the distillates were as follows: EtOH (99.9%), 2-PrOH (99.8%), and 2-PeOH (99.0%). The alcohols were stored over freshly dehydrated 4A-type molecular sieves in tightly closed containers.

#### 3.2.2. Hydrogen Acceptors

Commercial methyloxirane (puriss. Fluka AG, Buchs, Switzerland), *n*-butyloxirane (97%, Aldrich, Poznań, Poland), phenyloxirane (97%, Aldrich, Poznań, Poland), and 1,2-epoxycyclohexane (7-oxabiclo[4.1.0]heptane) (98%, Aldrich, Poznań, Poland) were purified by distillation under normal or reduced pressure (phenyloxirane), and the middle fractions were taken. After distillation, their purities, determined by GC, were as follows: 99.5, 98.6, 97.4, and 98.5%, respectively.

#### 3.2.3. GC Standards of Reaction Product

Synthesis of 1-(2-pentyloxy)-propan-2-ol: The synthesis was performed according to the procedure given in the literature [46]. In brief, 6.4 g (110 mmol) of methyloxirane were added to a solution of 1 g of NaOH in 48.4 g (550 mmol) of 2-pentanol kept at 388–390 K for 1 h. After 12 h of boiling, the mixture yielded 3.54 g of 1-(2-pentyloxy)-2-propanol, yield 24.0%.

Synthesis of 2-phenyl-2-(2-pentyloxy)-ethan-1-ol: The synthesis was carried out according to the procedure given in [47]. Benzoic acid (12.2 g, 100 mmol) and 2-pentanol (24.6 g, 282 mmol) were added to 6.0 g (50 mmol) of phenyloxirane. The solution was heated to reflux for 3 h, cooled, washed with a NaHCO_3_ solution and then with water. The layers were separated, and the aqueous layer was extracted with diethyl ether. The organic phases were combined and dried over anhydrous MgSO_4_. The volatiles were evaporated on a rotary evaporator. The crude product was distilled under reduced pressure, collecting the main fraction at 461–463 K/20 hPa, yield 2.3 g (23%).

### 3.3. Activity Measurements

Regardless of the mode of reaction, the reaction products were analyzed using an HRGC KONIK (Barcelona, Spain) gas chromatograph equipped with a TRACER WAX capillary column (length 30 m, 0.25 mm i.d.) and a flame ionization detector. The compounds were identified by GC-MS (HP-6890N with a 5973N mass detector (Agilent, Santa Clara, CA, USA)).

#### 3.3.1. Liquid-Phase Catalytic Activity Measurements

A weighed sample of magnesium oxide (250 ± 5 mg) was introduced into a one-piece cylindrical glass reactor, equipped with a condenser, followed by a magnetic bar, the hydrogen donor, hydrogen acceptor (usually 5 mmol), and *t*-butylbenzene as an internal standard. The reactor was heated by a silicon oil bath to a temperature 20 degrees higher than the boiling point of the donor to ensure ebullition of the entire solution. Samples of post-reaction mixtures were first centrifuged in order to separate the catalyst and then analyzed by the GC.

#### 3.3.2. Vapor-Phase Catalytic Activity Measurements

A weighed sample of magnesium oxide (250 ± 5 mg) was placed in a fixed-bed tubular glass reactor in a stream of nitrogen (50 cm^3^ min^−1^). After heating the reactor in the electric furnace to the set temperature, the reactant mixture containing *t*-butylbenzene was dosed using a microdosing pump with a Liquid Hourly Space Velocity (LHSV) of 3 cm^3^ per 1 g of catalyst per hour. The reaction products were collected in a receiver cooled to 273 K in an ice-water bath or, in the case of methyloxirane, to 243 K in a dry ice–2-propanol bath. The post-reaction mixture obtained during the first 60 min of the reaction was discarded. Samples were taken within the next 30 min of the reaction, and their composition was determined by gas chromatography.

## 4. Conclusions

Magnesium oxide was an active catalyst for the transfer hydrogenation carried out in the vapor phase in the temperature range of 473–673 K from alcohols (EtOH, 2-PrOH and 2-PeOH) to selected representatives of oxirane derivatives (methyloxirane, *n*-butyloxirane, phenyloxirane, and 1,2-epoxycyclohexane). The most important conclusions resulting from the research are as follows:

- In the case of catalytic hydrogen transfer to phenyloxirane from ethanol and 2-propanol, highly regioselective (100%) hydrogenation of the oxirane ring was observed to form 2-phenylethanol.
- In hydrogen transfer to methyl-, n-butyl-, and phenyloxirane, the dominant product is always the primary alcohol. The regioselectivity to this alcohol is 93, 73, and 100% for these oxiranes, respectively.
- 1,2-epoxycyclohexane reacts with alcohols to undergo a series of substrate isomerization and hydrogen transfer reactions. In the latter reaction, the highest yields of 22% for cyclohexanol and 14% for cyclopentylmethanol were achieved at 623 K.
- In the reaction with n-butyloxirane and 1,2-epoxycyclohexane, 2-propanol was a more reactive hydrogen donor than ethanol, but not in the case of phenyloxirane.
- In the liquid-phase mode of reaction, no CTH of the studied oxiranes has been observed. For phenyloxirane, the reaction with a hydrogen donor yielded 2-phenyl-2-(2-pentyloxy) ethanol, which is a product of an addition reaction.

## Figures and Tables

**Table 1:** Vapor-phase transfer hydrogenation of *n*-butyloxirane with 2-propanol in the presence of magnesium oxide as the catalyst. Influence of donor–acceptor molar ratio on the composition of the post-reaction mixture.

D/A	T [K]	Conv. [%]	Yields of Products ^1^ [%]			
			Me-CO-*n*-Bu	2-HeOH	1-HeOH	Others
3	523	1	0	tr	1	tr
	573	4	0	1	2	1
	623	38	12	6	13	7
	673	84	12	18	43	11
6	523	2	0	1	1	tr
	573	7	0	3	3	1
	623	49	10	12	21	6
	673	84	15	19	42	8
9	523	2	0	1	1	tr
	573	7	0	3	4	tr
	623	39	10	10	17	2
	673	91	14	20	54	3
6 ^2^	523	2	0	tr	1	1
	573	6	0	1	3	2
	623	23	12	2	5	4
	673	45	17	6	13	9

^1^—Me-CO-*n*-Bu = 2-hexanone; 2-HeOH = 2-hexanol; 1-HeOH = 1-hexanol; ^2^—ethanol as hydrogen donor.

**Table 2:** Time-on-stream test of vapor-phase transfer hydrogenation of *n*-butyloxirane with 2-propanol in the presence of magnesium oxide as the catalyst. D/A = 6, T_R_ = 673 K.

Time [h]	Conv. [%]	Yields of Products ^1^ [%]			
		Me-CO-*n*-Bu	2-HeOH	1-HeOH	Others
0.5	87	15	22	40	10
1.0	80	12	19	43	6
2.0	75	11	18	41	5
3.0	74	11	16	44	3
6.0	68	7	13	44	4
3.0 ^2^	62	7	11	41	3
6.0 ^2^	58	6	10	39	3

^1^—Me-CO-*n*-Bu = 2-hexanone; 2-HeOH = 2-hexanol; 1-HeOH = 1-hexanol; ^2^—repeated test with the spent catalyst.

**Table 3:** Liquid-phase transfer hydrogenation of phenyloxirane with 2-pentanol in the presence of magnesium oxide as the catalyst. D/A = 6, T_R_ = 392 K.

Time [min]	Conv. [%]	Yields of Products [%]			
		PhCH_2_CHO	2-PhEtOH	(RO)(Ph)EtOH ^1^	Others
30	17	12	0	5	tr
60	21	11	0	9	1
120	24	11	0	12	1
180	30	11	0	17	2
360	35	10	0	23	2

^1^—2-phenyl-2-(2-pentyloxy) ethanol.

## Data Availability

All acquired data is available in the paper.
